# Gut-Derived Sterile Inflammation and Parkinson's Disease

**DOI:** 10.3389/fneur.2022.831090

**Published:** 2022-03-29

**Authors:** Kathleen M. Shannon

**Affiliations:** Department of Neurology, School of Medicine and Public Health, University of Wisconsin, Madison, WI, United States

**Keywords:** Parkinson's disease, gut-brain axis, sterile inflammation, lipopolysaccharide, toll-like receptors, neuroinflammation

## Abstract

The etiology of Parkinson's disease (PD) is unknown, but evidence is increasing that there is a prominent inflammatory component to the illness. Epidemiological, genetic, and preclinical evidence support a role for gut-derived sterile inflammation. Pro-inflammatory bacteria are over-represented in the PD gut microbiota. There is evidence for decreased gut barrier function and leak of bacterial antigen across the gut epithelium with sub-mucosal inflammation and systemic exposure to the bacterial endotoxin lipopolysaccharide. Preclinical evidence supports these clinical findings and suggests that systemic inflammation can affect the CNS through vagal pathways or the systemic circulation. We will review recent preclinical and clinical evidence to support this mechanism and suggest possible treatments directed at the gut-brain axis.

## Introduction

The central nervous system (CNS) trail of Parkinson's disease (PD) is defined by α-synuclein (AS) aggregates in cell bodies and neuritis (1). The etiology of this synucleinopathy remains obscure, but is generally ascribed to a confluence of age, genetics and environmental risks (2). Age, genetic, and environmental actors have a nexus with inflammation (3), and it is now accepted that CNS inflammation plays a significant role in the progression of PD. Microglia are the resident myeloid cells in the CNS. In the presence of certain stimuli, including products of neurodegeneration, microglia undergo a morphological change into an “activated” state and unleash an inflammatory cascade (4). The first reports of activated microglia in association with synucleinopathy in PD decedents date back to Foix's description in 1925 (5), and were confirmed by McGeer's report in 1988 (6). In 2003, Imamura found evidence of markers of activated microglia and increased tumor necrosis factor alpha (TNFα) in PD brains (7). More recently, PET imaging using PK-11195 has uncovered evidence of microglial activation in living brain (8). Neuroinflammation as a response to neurodegeneration is settled science, but what is less well-enshrined is that systemic sterile inflammation may be important in the genesis of neurodegeneration itself.

## The Enteric Nervous System and Gut-Brain Axis

The gut-brain axis is a bidirectional system that comprises the enteric nervous system (ENS), the autonomic nervous system, the central nervous system and hormones of the hypothalamic-pituitary-adrenal axis (9). The ENS is complex, with a tripartite submucosal plexus structure, and 2 additional plexuses, myenteric, and Auerbachs. Enteric glial cells (EGC) outnumber enteric neurons by a factor of about 7, and are critically responsible for the development, growth and maintenance of gut homoeostasis (10). EGCs have antigen-presenting capacity and form a vital part of the innate immune system. The ENS functions autonomously, but has rich connections with the CNS through the vagus nerve and spinal cord. The intestinal microbiota are essential for normal development and maintenance of EGCs and communicate with the host via action at pattern recognition receptors as well as secretion of substances such as short-chain fatty acids, neurotranmitters and neurotransmitter precursors (9).

## The Role of Inflammation in the Genesis and Progression of PD

### Sterile Inflammation and the Gut

Sterile inflammation is defined as inflammation in the absence of a pathogen. A number of stimuli, including those caused by trauma, ischemia, or environmental instigators produce damage-associated molecular patterns (DAMPs). These act at innate immune receptors (toll-like receptors (TLR), RIG-1-like receptors (RLS) and Nod-like receptor family, and pyrin domain containing 3 (NLRP3, which respond to pathogen-associated molecular patterns (PAMPs) and damage-associated molecular patterns (DAMPs). TLRs activate myeloid-differentiation factor-88—dependent and—independent signaling pathways that unleash the production of inflammatory cytokines. These include tumor necrosis factor alpha (TNF-α), interleukin (IL)-1, IL-6, IL-8 and IL-12). DAMPs also activate the NLRP3 inflammasome, activating caspase-1, resulting in the production of IL-1β and IL-18. Increased levels of inflammatory cytokines have been documented in the brain and cerebrospinal fluid in PD (11–13), but also in the blood (14), suggesting an important role for systemic inflammation in PD. These cytokines variably include TNFα, IL-6, IL-1β, CRP, IL-10, interferon-γ, IL-1β, and IL6.

Interest in the gut as a possible instigator in the inflammatory process arises from several observations. The gut surface area, estimated between 32 and 300 M^2^, is the largest body surface exposed to the environment. The intestines form a critical barrier to penetration of pathogens and their antigens, and make up the majority of the surface related innate immune system. In close proximity to the intestinal epithelium are millions of neurons in direct contact through the vagus nerve with the brainstem and through spinal nerves to the spinal cord. Commensal bacteria in the gut, the gut microbiota, include some 100 trillion micro-organisms (9). A change in gut microbiota, dysbiosis, can be associated with systemic or neurological disease (9). Epidemiological studies suggest a significantly increased risk of PD in people with inflammatory bowel disease, particularly when the onset is at an older age, and the risk is significantly reduced with therapies targeting TNF-α (15, 16). Prodromal PD is characterized by anosmia and constipation, suggesting a nasal or oral portal of entry of a potential pathogen (17). There are correlations in elderly populations between prodromal PD risk markers and dysbiosis (18). In 2006, Braak proffered that autopsy studies suggested an inciting pathogen caused AS aggregation that spread in a prion-like fashion from the enteric nervous system, entering the CNS through the vagus nerve at its dorsal motor nucleus (19). This hypothesis was buoyed by a number of studies showing AS pathology in biopsied or excised tissue in living PD subjects (20–24) and by preclinical studies showing a potential role of pathological spread of synuclein aggregation to contiguous neurons (25–27). However, this proposed mode of PD genesis has been undermined by inconsistencies in study findings, similar AS immunostaining in control subjects and the failure to find AS aggregates isolated to the GI tract in more than 400 autopsies (20, 28–30). An alternative hypothesis for a role of the gut in the genesis of PD is that local inflammatory influences in the gut drive systemic inflammation that then spreads to the CNS. Our own studies support the theory that altered gut microbiota degrade gut barrier function, driving gut inflammation and systemic exposure to the bacterial endotoxin lipopolysaccharide (LPS). We believe that the LPS produced in this gut-derived sterile inflammation process drives a systemic innate immune process that accesses the central nervous system to produce a CNS synucleinopathy that itself sustains a neuroinflammatory process. This review will summarize the research findings that support this concept, discuss future research questions, and speculate on future therapeutic strategies.

### Microbiota Changes

The commensal bacteria of the gut play essential roles in the development and maintenance of the intestinal epithelium, the control of gastrointestinal motility, and control of the intestinal barrier. The bacteria also protect the intestinal lumen from pathogenic bacteria (31). The human gut harbors more than 1,000 species-level phylotypes of bacteria (32). Bacteria in the phyla Firmicutes and Bacteroidetes are the most prevalent gut commensals, but representatives from Actinobacteria, Proteobacteria and Verrucomicrobia are also common (33). There are a number of things that influence the makeup of the GI microbiota including age, genetics, environment, antibiotic use, and diet (34). There is evidence that short chain fatty acids, produced by healthy gut microbiota metabolism of dietary fiber, are important in the maintenance of normal function of the gastrointestinal tract as well as general health (35). Most studies of microbiota now employ molecular detection methods, predominantly those that require sequencing of the hypervariable regions of the 16S ribosomal RNA genes, metagenome sequencing, or whole bacterial meta-transcriptome sequencing (34).

Our group studied the fecal microbiota of 9 early PD subjects who did not take dopaminergic medications, 29 levodopa-treated PD subjects, and 34 healthy controls (HC) using high throughput rRNA gene amplicon sequencing of the V4 variable region of the 16S ribosomal RNA gene. PD subjects had greater α-diversity. At the phylum level, Bacteroidetes, Proteobacteria, and Verrucomicrobia were higher, and Firmicutes were lower in PD samples. At the genus level, putative anti-inflammatory bacteria from the genera *Blautia, Coprococcus* and *Roseburia* were under-represented in PD feces, and pro-inflammatory bacteria (*Akkermansia, Oscillospira*, and *Bacteroides*) were over-represented (36). There have now been nearly 2 dozen studies of gut microbes in PD, and most have concluded that pro-inflammatory bacteria are over-represented in the PD gut, though there were significant differences among the studies. Other studies have found reductions in the prevalence of bacteria from the Prevotellaceae and Lachnospraceae families with increases in representatives of the Verrucomicrobiaceae and Lactobacillaceae families (37–49). In one study, the relative abundances of 4 families (*Prevotellaceae, Lactobacillaceae, Bradyrhizobiaceae, and Clostridialis Incertae Sedis IV*) identified PD cases with 47.2% sensitivity and 90.3% specificity (48). A recent meta-analysis reviewed 22 microbiota studies that used 16S ribosomal RNA-gene amplicon sequencing (50). In 10 of these, raw sequencing data were available and re-analyzed (36, 37, 40–42, 47, 48, 51–53). These 10 studies included 9 cohorts from six countries. The authors found that methodological differences (country of origin, selection of study participants, sample handling, sequencing platform, and selection of which variable region of the 16S rRNAgene to analyze) drove inconsistencies among the studies. However, there were significant differences in microbiota between PD and HC. The differences were considered robust, characterized by a decrease in the most abundant members of the population and an increase in abundance of normally less abundant species. *Roseburia, Fusicatenibacter, Blautia, Anaerostipes, and Faealibacterium* were less represented in PD samples. The most significant changes were predicted to result in reductions in stool short-chain fatty acids (SCFA), particularly butyrate. SCFAs have anti-inflammatory and anti-oxidant activities and are important in the regulation of intestinal and blood brain barrier permeability through effects on tight junction proteins (54). Standardized processes for future studies were recommended (50). In addition to associations with the disease, some studies have found that gut microbiota changes may be associated with disease duration or scores on motor, non-motor, or behavioral outcomes measures (34, 40, 48, 52, 55–57). Predictive metagenomics studies suggest changes in the micriobiome favor increased pathogenicity, reduced normal metabolic functions, and increased LPS synthesis (36).

The potential importance of fecal microbiota changes in the genesis of PD is supported by preclinical studies. In preclinical models, behavioral, and pathologic markers of parkinsonism in mice engineered to over-express AS are significantly reduced in germ free mice, which lack intestinal microbes (58, 59) and a more robust parkinsonism is seen when the gastrointestinal tract is repopulated with dysbiotic microbiota (59). Fecal microbiota transplantation protects mice from rotenone induced parkinsonism (60). Stress-induced gut dysfunction provoked a pro-inflammatory gut dysbiosis, increased systemic LPS, activation of brain microglia, death of dopaminergic cells in the substantia nigra, and dopamine deficiency in oral rotenone-induced parkinsonian mice (61). Intraperitoneal MPTP injections produced parkinsonism in mice, accompanied by abnormal intestinal microbiota. Transplantation of the dysbiotic microbes into wild type mice caused the recipients to become parkinsonian (62).

### Intestinal Leak in PD

The intestinal barrier is a single epithelial layer lining the GI tract. The barrier regulates the transport of luminal contents across the intestinal wall by paracellular and transcellular transport (63). Paracellular transport of ions and water-soluble solutes is controlled by tight junction proteins (TJP), principal among them the claudin family, occludin, junctional adhesion molecules, cytosolic scaffold proteins, and intracellular zona occludens. Loss of integrity of the intestinal barrier produces a leaky gut, allowing access across the mucosa to bacterial antigens, including LPS.

**Figure 1:**
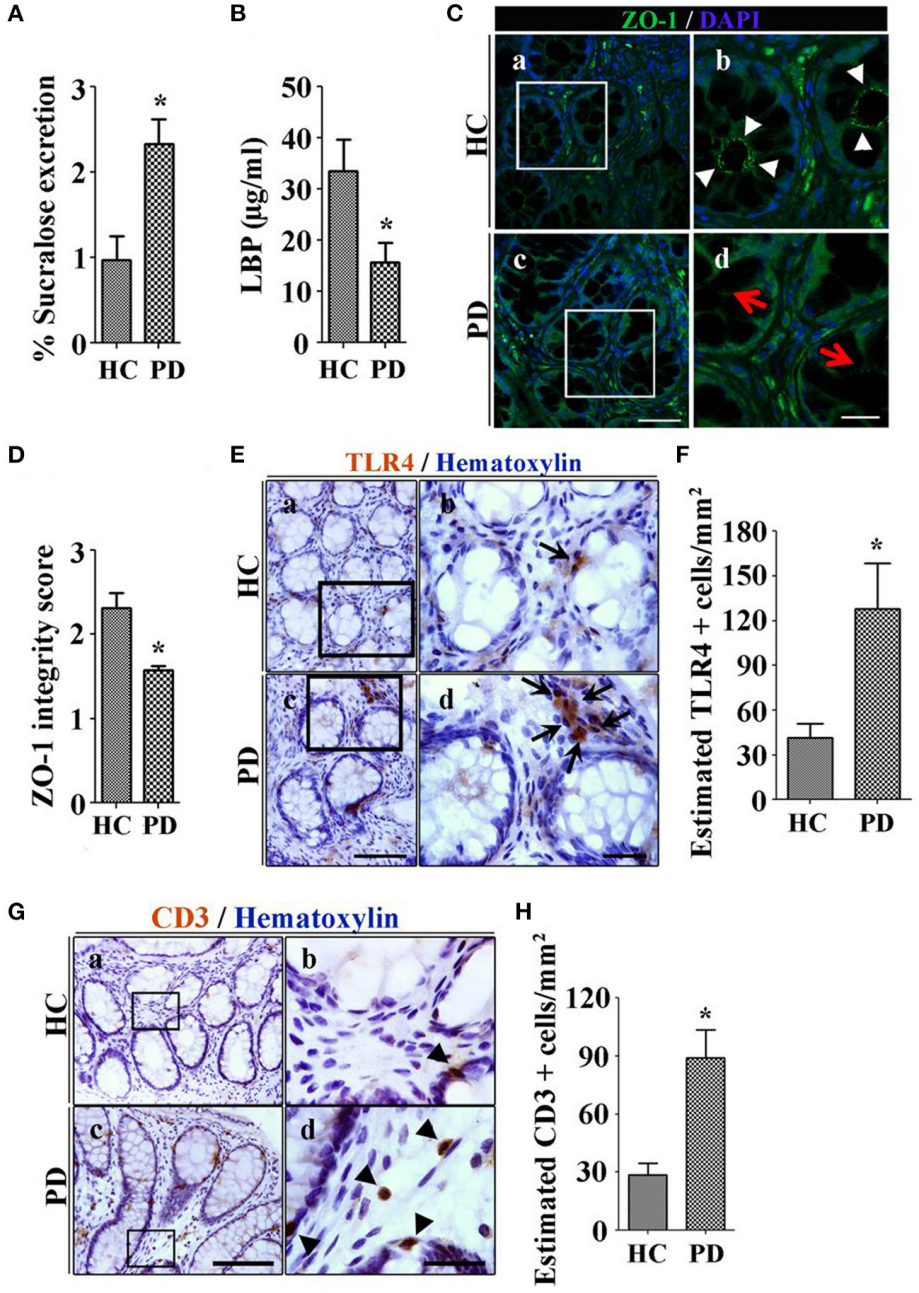
Patients with Parkinson's disease (PD) show increased colonic permeability and associated colonic inflammatory as well as immune markers in their biopsies compared with healthy controls (HC). **(A)** Per cent excretion of sucralose in the urine samples as an intestinal permeability marker. **(B)** Levels of LPS-binding protein (LBP) as a marker for systemic endotoxin in plasma samples. **(C)** Photomicrographs of immunofluorescence staining of tight junction protein zonula occludens 1 (ZO-1) in HC (a,b) and PD (c,d) colonic mucosa. **(D)** Integrity scoring for ZO-1 tight junction protein expression in colonic samples. **(E)** Photomicrographs of stained toll-like receptor 4 (TLR4)+ cells in lamina propria of the HC (a,b) and PD mucosa (c,d). **(F)** Estimated TLR4+ cells expressed as number/mm^2^. **(G)** Photomicrographs of stained CD3+ T cells in colonic mucosa of HC (a,b) and PD (c,d). **(H)** Assessment of CD3+ cells in lamina propria of colonic samples. Scale bars: C(c) = 50 μm and C(d)=20 μm, E(c)=75 μm and E(d)=25 μm, G(c)=40 μm and G(d)=25 μm. *P < 0.05, **p < 0.001, ***p < 0.0001. Data represent mean ± SEM.

The most common way to assess intestinal barrier function in man is measurement of the urinary excretion of ingested poorly absorbed sugars. Using this method, we studied intestinal permeability in nine subjects with early untreated PD. Subjects ingested an oral load of 2 g mannitol, 7.5 grams lactulose, 40 g sucrose and 1 g sucralose after an overnight fast. Urine was collected in two 12-h aliquots and the sugar content was analyzed using gas chromatography and expressed as percent of ingested load. PD subjects excreted significantly more sucralose over 24 h than controls, suggestive of increased total intestinal permeability. Increased permeability was associated with evidence of *E. coli* antigen and inflammation in mucosal biopsy specimens (64). Another way to assess intestinal leak is the measurement of calprotectin, alpha-1-antitrypsin and zonulin, all markers of increased intestinal permeability in stool specimens. Schwiertz et al. found that all 3 fecal markers were significantly elevated in PD compared to controls (65), and Aho et al. found similar results for calprotectin and zonulin (66, 67). However, using a different technique to measure intestinal leak, Clairembault et al. measured flux of sulfonic acid for paracellular and horseradish peroxidase for transcellular flux in an using chamber experiment. There was no evidence of increased intestinal permeability in PD in this study, though subjects with PD did show evidence for inflammation in the colonic epithelium (67). To explore the mechanism of intestinal leak, we assessed the integrity of the TJP in PD subjects using immunocytochemistry, and found decreased integrity of ZO-1 in PD (68) (see Figures 1C,D). This finding has been confirmed in other studies (67).

### Lipopolysaccharide

Lipopolysaccharide (LPS) is an endotoxin specific to gram-negative bacteria (69). LPS binds to lipopolysaccharide binding protein (LBP) in the serum and is transferred to the CD14 receptor, then transferred to MD2 protein which interacts with toll-like receptor 4 (TLR-4). LPS acts at and incites an inflammatory response via recruitment of downstream adapters ultimately resulting in the production of inflammatory cytokines including TNF-α, IL-1, IL-6, and IL-8 (70). Systemic exposure to LPS occurs through the gut. Systemic LPS is involved in catastrophic acute inflammatory disorders such as sepsis, but it has also been linked to systemic and organ-specific inflammatory states such as the metabolic syndrome, obesity, deranged glucose metabolism, inflammatory bowel disease (71), fatty liver, and kidney and heart disease (72). LPS disrupts intestinal TJPs and causes intestinal leak (39), and increases intestinal AS expression (73). LPS was first linked to PD when accidental exposure of a laboratory worker resulted in the development of parkinsonian symptoms with imaging confirmation of nigral damage (74). There is evidence in PD of access of LPS to the intestinal wall and to the systemic circulation. At the level of the intestinal wall, we have demonstrated upregulation of the TLR-4 receptors in the PD gut submucosa (68) (see Figure 1E). Low levels of LBP in plasma, a marker of increased systemic LPS, are seen in PD subjects (see Figure 1B) (64, 75).

Intraperitoneal injections of LPS in gravid rats leads to a reduced number of dopamine neurons in the substantia nigra of their offspring, and enhanced sensitivity to 6-hydroxydopamine induced parkinsonism compared to offspring of control animals (76). LPS has been used to model PD when administered into the substantia nigra, striatum, globus pallidus, and cerebral ventricles (77). Systemic (intraperitoneal or intranasal) LPS can be used to model manifest and prodromal PD in mice. Single exposure to high systemic doses and repeated administration of lower doses can produce a delayed and progressive PD phenotype in mice with behavioral changes, synucleinopathy in the enteric nervous and central nervous systems and increased intestinal permeability (77). In preclinical models, LPS is known to disrupt blood brain barrier function, a property is enhanced by AS expression (78). In mice, LPS alters the fibrillary strain of AS and changes its aggregation kinetics. This altered strain has the property of self-renewal and can be used to model synucleinopathy (69). Once the inflammatory process has entered the CNS, it is self-sustaining. AS is a DAMP that acts at TLRs (TLR2 and possibly TLR4) (79) inciting pro-inflammatory influences in micgoglia (79, 80).

### Potential Mechanisms of Gut-Derived Sterile Inflammation in the Genesis of PD

It would be premature to say that the mechanisms by which gut-derived sterile inflammation influences the genesis of PD are completely understood. In the gut, it seems well-established in PD that gut dysbiosis is present, is associated with intestinal leak and penetration of bacterial antigens including LPS into the intestinal wall and production of local inflammatory mediators, induced by action at TLRs. This inflammatory trigger may produce a systemic inflammatory process with contributions from both the innate and adaptive immune systems (9). Systemic inflammation may alter the permeability of the blood brain barrier and allow access of systemic immune cells or inflammatory cytokines to the CNS. This induces a neuroinflammatory process with microglial activation and AS aggregation (81). Once synucleinopathy begins, it is a self-sustaining process in which synuclein acts as a DAMP, stimulating TLRs and causing cytokine release (54), with the potential to spread through a network of contiguous neurons. An alternative explanation, driven in part by the Braak hypothesis is that gut inflammation drives AS aggregation in the intestinal submucosa, with prion-like spread through the vagus nerve to its dorsal motor nucleus, from which aggregates spread in a caudal to rostral direction producing prodromal lower brainstem signs, then specific motor signs of dopamine deficiency and ultimately complications related to spread to non-dopaminergic brain regions (19). A nagging question in this discussion relates to whether it can definitively be stated that these intestinal changes are a potential cause rather than an effect of PD. One obstacle to resolving this question in human studies is the inability to identify PD during the early stages of CNS disease and more importantly, during the peripheral prodromal state. It has been estimated that the onset of dopaminergic degeneration predates clinical parkinsonism by five or more years, and peripheral prodromal symptoms such as constipation may begin 13 or more years before diagnosis. Thus, finding the origins of PD will require its identification perhaps decades before it reaches a stage that clinical recognition is possible. While there are now both a defined prodromal phenotype and accompanying diagnostic criteria (17), the sign that best predicts the prodromal state is the presence of rapid-eye-movement sleep behavioral disorder, which occurs after the CNS is breached by the destructive process. Viewed from the perspective of therapeutics, agents that target peripheral inflammation may be ineffective once self-sustaining neuroinflammation is established.

In the same way that changes in intestinal function are associated with non-neurological disorders that share systemic inflammatory drivers, these changes are not specific among neurodegenerative diseases for PD. For example, studies in another synucleinopathy, multiple system atrophy also show evidence for an inflammatory dysbiosis (82). Changes as described in this review have also been reported in neurodegenerative diseases that are not synucleinopathies, such as Alzheimer's disease (83). One explanation for this observation may be that the type of proteinopathy that develops in a given subject is related to the intersection of genetic susceptibility with neuroinflammation. On a hopeful note, to the extent that treatments can be developed to intervene in sterile inflammation-related synucleinopathy, or other proteinopathies, these may be applicable across neurodegenerative phenotypes (83, 84).

### Toward Treatment

Despite these limitations, attention is turning toward potential therapies. Dietary changes targeting the gut-brain axis are a tempting prospect. Diets rich in fish and healthy snacks reduced LPS activity in serum in a non-PD population (85), and reduced incidence of PD has been linked to adherence to a Mediterranean diet (86). Such diets are rich in fruits, vegetables, fiber, and olive oil, with reduced reliance on dairy products and red meat. They are associated with health gut microbes, SCFA production, anti-inflammatory and anti-oxidant activities. There are no published studies of the effects of diet on progression of extant PD.

Living bacteria, in the form of probiotics, can be delivered orally via dietary supplements, and these can have a salutary effect on inflammatory bowel disease, such as ulcerative colitis. A pilot placebo-controlled study of probiotics in 50 PD patients showed decreased expression of genes associated with inflammation (IL-1, IL-8, and TNFα) (87), and another suggested motor improvements (88). Further study of probiotics in PD will be necessary to define the optimal bacterial composition of such supplements.

Moderate-intensity exercise reduced systemic inflammation and decreased TNFα in PD subjects (89). Despite a recent proliferation of exercise studies in PD, definitive proof of benefit on disease progression remains elusive.

Fecal microbial transplant has the potential to restore the gut microbiota to normal (90). Although studies have established at least some potential to modify intestinal and systemic inflammation, well-designed studies in PD have not been performed.

At the time of this writing, clinicaltrials.gov listed 378 interventional clinical trials in PD. Among these, only 8 targeted the gut, including studies or prebiotic fiber supplements, probiotics, anti-inflammatory diet, nutritional supplement and fecal transplant (clinicaltrials.gov).

## Author Contributions

This article was written solely by KS, but reflects collaborative work with a number of investigators, principally among them from Rush University Medical Center (AK, CF, JK), University of Chicago (HD, PP-P), California Institute of Technology (TS, SM) University of Gainsville, Florida (MT), and Emory University (MH).

## Conflict of Interest

The author declares that the research was conducted in the absence of any commercial or financial relationships that could be construed as a potential conflict of interest.

## Publisher's Note

All claims expressed in this article are solely those of the authors and do not necessarily represent those of their affiliated organizations, or those of the publisher, the editors and the reviewers. Any product that may be evaluated in this article, or claim that may be made by its manufacturer, is not guaranteed or endorsed by the publisher.
